# Observing the brain pulsating

**DOI:** 10.1007/s00508-021-01980-2

**Published:** 2021-11-19

**Authors:** Alexandros Andrianakis, Peter Valentin Tomazic

**Affiliations:** Department of Otorhinolaryngology, Medical University of Graz, Auenbruggerplatz 26, 8036 Graz, Austria

A 75-year-old man presented to the otorhinolaryngology outpatient clinic with a 3-month history of nasal airway obstruction. With anterior rhinoscopy, a livid-colored capillary expansion obstructing the whole nasal cavity was detected. A biopsy specimen was diagnosed as a capillary hemangioma, which was later resected endoscopically in sano. An additional finding of the initial physical examination was a frontal pulsating skin indentation with a central scar (Fig. 1; Video 1, Supplementary material). The patient reported a car accident that occurred 30 years ago as the origin of this indentation. No further information from the patient’s history or the medical record was available. Magnetic resonance imaging (MRI) of the face and brain was performed to assess the extent of the nasal hemangioma and, unrelated to the intranasal expansion, an arachnoid cyst below the osseous defect of the left frontal bone was detected with subsequent dilatation of the left lateral ventricle (Fig. 2). The patient’s medical history revealed no predisposing diseases or other relevant medical conditions. The diagnosis of a secondary posttraumatic arachnoid cyst was established [1]. On follow-up MRI, this arachnoid cyst without clinical symptoms was stable and remains under surveillance. This is a rare case of a pulsating frontal bone defect due to secondary posttraumatic arachnoid cyst with extension into the lateral ventricle.

**Fig. 1:**
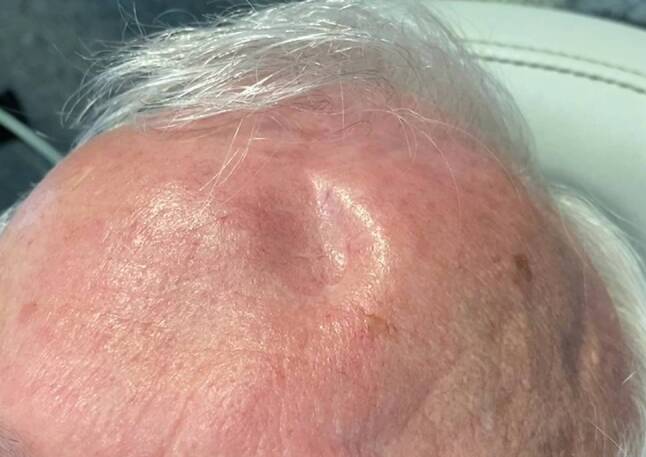
Frontal pulsating skin indentation with a central scar

**Fig. 2:**
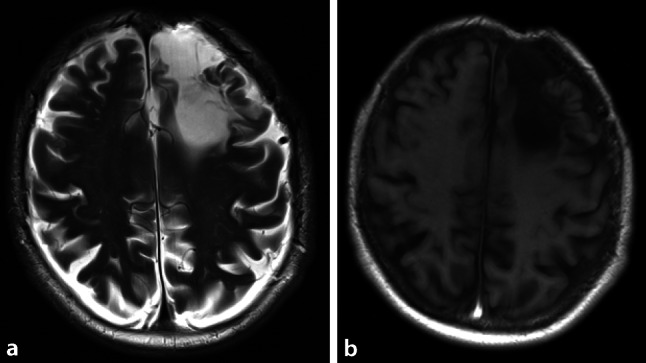
Secondary posttraumatic arachnoid cyst below the osseous defect of the left frontal bone with subsequent dilatation of the left lateral ventricle on cranial magnetic resonance imaging (**a** T2-weighted, **b** T1-weighted)

## Supplementary Information

Video of frontal pulsating skin indentation.
